# Accurate Fiducial Point Detection Using Haar Wavelet for Beat-by-Beat Blood Pressure Estimation

**DOI:** 10.1109/JTEHM.2020.3000327

**Published:** 2020-06-05

**Authors:** Muskan Singla, Syed Azeemuddin, Prasad Sistla

**Affiliations:** 1 Centre of VLSI and Embedded System TechnologyInternational Institute of Information TechnologyHyderabad500032India; 2 Care FoundationCare Hospital 194141 Hyderabad500034India

**Keywords:** Adaptive algorithm, blood pressure, detection algorithm, electrocardiography, feature extraction, photoplethysmography, regression analysis, signal processing, wavelet transforms

## Abstract

Pulse Arrival Time (PAT) derived from Electrocardiogram (ECG) and Photoplethysmogram (PPG) for cuff-less Blood Pressure (BP) measurement has been a contemporary and widely accepted technique. However, the features extracted for it are conventionally from an isolated pulse of ECG and PPG signals. As a result, the estimated BP is intermittent. Objective: This paper presents feature extraction from each beat of ECG and PPG signals to make BP measurements uninterrupted. These features are extracted by employing Haar transformation to adaptively attenuate measurement noise and improve the fiducial point detection precision. Method: the use of only PAT feature as an independent variable leads to an inaccurate estimation of either Systolic Blood Pressure (SBP) or Diastolic Blood Pressure (DBP) or both. We propose the extraction of supplementary features that are highly correlated to physiological parameters. Concurrent data was collected as per the Association for the Advancement of Medical Instrumentation (AAMI) guidelines from 171 human subjects belonging to diverse age groups. An Adaptive Window Wavelet Transformation (AWWT) technique based on Haar wavelet transformation has been introduced to segregate pulses. Further, an algorithm based on log-linear regression analysis is developed to process extracted features from each beat to calculate BP. Results: The mean error of 0.43 and 0.20 mmHg, mean absolute error of 4.6 and 2.3 mmHg, and Standard deviation of 6.13 and 3.06 mmHg is achieved for SBP and DBP respectively. Conclusions: The features extracted are highly precise and evaluated BP values are as per the AAMI standards. Clinical Impact: This continuous real-time BP monitoring technique can be useful in the treatment of hypertensive and potential-hypertensive subjects.

## Graphical abstract

We introduce a plug-integrate-play (PIP) conceptual model for an x-ray imaging system and implement it as an open hardware platform, SyncBox. Based on validation studies, we believe that SyncBox holds potential for introducing an open source hardware platform to x-ray equipment design.

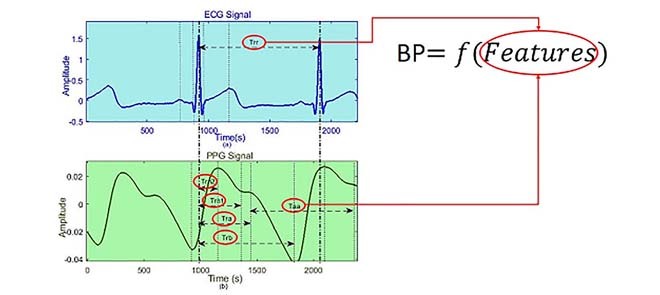

## I. Introduction

Hypertension has been termed as a silent killer because it adversely affects the body organs such as the brain and kidney, mostly without showing symptoms. It escalates the burden of heart disease, stroke, kidney failure, and premature death & disability [1]. As per the Global Health Observatory (GHO) data provided by the World Health Organization (WHO), raised blood pressure (BP), which is defined as 140 mmHg or higher for systolic blood pressure (SBP) and/or 90 mmHg or higher for diastolic blood pressure (DBP), affects 1.13 billion people worldwide [2], which is nearly grown twice since 1975 [3]. It is a leading risk factor for cardiovascular diseases and chronic kidney diseases [4], which are treatable in prior stages by continuous monitoring, by controlling the BP using drugs or by switching to a healthier lifestyle.

There are several techniques available for BP estimation, viz, auscultatory, oscillometric, finger cuff, tonometry, invasive arterial line, ultrasound, etc. The respective advantages and disadvantages have been summarized in [5]. Among these techniques, the auscultatory and oscillometric have been quite popular for ambulatory measurement, but they involve the inflation and deflation of the cuff which occludes the artery causing tissue damage and discomfort. Currently, for intermittent monitoring, inflation and deflation of the cuff are timely automated by an oscillometric system, but it not only induces pain but also the sampling frequency for measurement is too low to detect the fluctuations in BP. Hence, cuff-less BP measurement is essential for continuous BP monitoring [6], [7].

Cuff-less BP can be estimated using Pulse Wave Velocity (PWV) derived from the Pulse Transit Time (PTT), based upon the Moens-Korteweg (M-K) equation, as illustrated in (1). PWV is defined as the ratio of ’$d$’ and PTT, where ’$d$’ is the distance between heart and arterial site of measurement, and PTT is the time taken by the blood to flow between the two sites.

\begin{equation*} PWV=\frac {d}{PTT}=\sqrt {\frac {E h}{\rho D}} \tag{1}\end{equation*}

Equation (1) explains the dependency and relationship of PWV with physiological factors, that is, the density of blood ($\rho $), wall thickness ($h$), elastic modulus ($E$) and diameter of the artery ($D$) [8]. The equation for elastic modulus is given by,

\begin{equation*} E=E_{0} e^{\gamma P} \tag{2}\end{equation*}

Among the four factors, elastic modulus ($E$) varies exponentially with BP as shown in equation (2), where ($E_{0}$) is the elastic modulus at zero pressure, ($P$) is the pressure, and ($\gamma $) is the coefficient depending on various parameters of the vessel, age, body mass index (BMI), etc. [9].

PTT is calculated as the time difference of the distal waveform foot from the proximal foot. Strategy for PTT extraction is not absolute, viz, PTT can be the time duration between the foot of Impedance Cardiography (ICG) signal and photoplethysmograph (PPG) signal. PTT can also be extracted out of two PPG signals acquired from two different arterial sites [10]–[12]. Pulse arrival time (PAT) is the sum of PTT and pre-ejection period (PEP) [7], [13]. PAT is the time delay between electrical and the distal pulse i.e. occurrence of R peak and the systolic peak of a concurrent electrocardiogram (ECG) and PPG signal respectively [6], [10], [14]–[19].

However, PAT cannot be solely enough [13]. This is because, from (2), it can be realized that multiple features influence the elasticity, thus affecting PWV, inhibiting the use of PAT as an independent variable for BP estimation [6], [13], [14].

To circumvent this inaccuracy in BP estimation, extra features in addition to PAT have been used. Estimation has been done using PPG Intensity Ratio (PIR) in combination with PAT [14], [20] or by performing PWA on a single PPG signal [21], [22]. Arterial BP oscillates due to respiration which can be tracked using PTT, but for the fluctuation due to vasomotor tone, PIR is considered. Hence, to improve the accuracy of the estimation at low frequency, PIR is taken into account by Ding *et al.* [14], [20]. Reference [13] proposed a model based on Impedance Photoplethysmography (IPG) and PTT to increase the accuracy, which takes input from IPG to calculate the impedance where the minimum and maximum impedance were used to estimate SBP and DBP respectively. Most of the above-stated works involve intermittent calibration using the existing technologies as reference. Therefore, it would be safe to say that, for more accuracy and robustness, the feature dimension can be increased. This improvement in the accuracy of detection with the use of multiple features extracted out of ECG and PPG signal can also be witnessed in [6], [10], [23].

The precise detection of the wave points such as R peak, PPG peak, etc., and explicit feature extraction plays a significant role in the estimation of BP. The approach to detect the above-stated wave points involves thresholding and derivative techniques [12], [24]. These techniques can detect R peak as well as PPG peak with acceptable precision, however, it is not capable enough to detect the rest of the wave points. Hence, wavelet transformation has been used, which explores the information present in various frequency components of the signal and also provides the respective time-domain information.

Haar wavelet, due to its reduced computation complexity and ability to detect sudden changes, has been used in this paper to detect the required wave points in ECG and PPG signal as discussed in section II-B.

For the development of an independent feature vector, some of the above-stated research works involve the extraction of features from an isolated pulse of the physiological signal [12], [16], [24], [25]. Since the reference BP is measured using cuff based techniques, which provides BP after occlusion for about a minute, hence it is not a surrogate for beat by beat pressure. As a result, features from a single pulse may result in incorrect estimation. Therefore, it is essential to consider continuous signals for feature extraction and BP estimation. But, the extraction from continuous signals has various challenges. Due to the mental and physical activities of the subject, the cycle period of these physiological signals vary. To encounter the heart rate and pulse rate variability in the process of feature extraction, Adaptive Window Wavelet Transformation (AWWT) technique is proposed. AWWT differentiates each beat while ensuring the presence of a complete cycle, and extracts the features supplementing PAT with certainty as discussed in section II-C and II-D.

In the proposed work, we detect multiple points on continuous PPG, as well as ECG signals to develop a feature vector of 32 features. These features have their physiological significance and are highly correlated, hence improves the accuracy of the estimation. These features are discussed in section II-F.

The training can be done using various regression models such as linear regression (used most often) [23], [24], [26], non-linear regression models [27] and other machine learning techniques such as neural networks [11], [21]. The proposed model is trained using a log-linear regression analysis. The significance of using this training technique is discussed in section II-G.

These training techniques require extensive and concurrent ECG and PPG data sets for appropriate modeling of BP and to establish the fidelity of the proposed algorithm. In most of the studies, the MIMIC database is used, however, there are some limitations with the concurrence of the ECG signal, PPG signal, and the reference BP in the database. Therefore, some research works developed a database to establish the relation of BP with PAT and other related features. Here, in this research which is an extension of [28], we collected simultaneous ECG and PPG data for 171 human subjects, from the population of the age group between 22 years to 55 years as discussed in section II-A. The results obtained after processing and analyzing the data are shown in section III. Finally, the entire work is summarized in section IV.

## II. BP Estimation Method

This section focus on the procedure of data collection, data analysis, feature extraction and finally training a regression model using the feature vector. The data required should be highly precise and reliable as it forms the foundation for each succeeding step. The following subsection explains the process of data collection.

### A. Data Collection

We collected digital and simultaneous data for ECG and PPG signals along with reference BP using the Vios monitoring system (VMS) from CARE Hospital, Hyderabad, India. VMS has been cleared by the Food and Drug Administration (FDA) [29]. It is used for monitoring ECG, pulse rate, heart rate, SPO_2_, and temperature. ECG limb leads (I, II) are sampled by TI ADC ADS1292. Pulse Ox analog signals (LEDs) are sampled by TI ADC AFE4490. Both the ADCs are driven by the same clock. It also allows the external input of Non-Invasive Blood Pressure (NIBP) using Omron’s digital sphygmomanometer.

Data was collected for 171 human subjects for about 5–7 minutes each and NIBP was measured alongside. All the subjects gave their informed consent for participation and the study has been approved by the ethics committee of CARE Hospital. The experiment was conducted as per AAMI sphygmomanometer guidelines [30]. The BP values distribution within the database is described in Table 1. The variation in the database has been maintained as per [31]. Hence, this study is focused on the estimation of normal to hypertensive patients.

**TABLE 1:** Subjects Lying in Various BP Ranges

BP classification	SBP (mmHg)		DBP (mmHg)	Subject count
Normal	< 120	and	< 80	40
Prehypertension	120-139	or	80-89	87
Stage 1 hypertension	140-160	or	90-100	29
Stage 2 hypertension	> 160	or	> 100	11

The algorithm was developed using MATLAB 2019. The ECG and PPG data obtained from VMS are sampled at 200 samples/sec and 75 samples/sec respectively. Though the least common multiple of sampling rates is 600 samples/sec, this signal is interpolated by the factor of 6 to obtain 1200 samples/sec. This is because, in the proposed algorithm, wavelet decomposition is performed up till the fifth level and at each level, the samples within the window reduce to half. For performing Wavelet transform modulus maxima (WTMM), the input must be at least 128 samples. The wave points required for the feature vector generation are detected using wavelet transformation, which has been discussed below.

### B. Wavelet Transformation

Physiological signals such as ECG and PPG signals are composed of multiple frequency components. By distinguishing these frequencies, various wave points can be identified on the signals. To achieve this, interpretation should be done in the frequency domain, such that we can have concurrent information about the time domain as well. Fast Fourier Transformation (FFT) is capable of performing frequency domain analysis, but it cannot provide the required time-domain information. An alternative could be the Short Frame or Short-time Fourier Transformation (SIFT), which may allow space-frequency localization, but Wavelet Transformation (WT) enables effective extraction of information from both frequency and time domain. It can also eliminate noise and artifacts. In the case of WT, a signal can be represented using various analyzing functions apart from a sinusoidal signal. Hence, WT can be used for wave point detection within ECG and PPG [25], [32]–[37]. Discrete Wavelet Transformation (DWT) has been used here.

**FIGURE 1.**
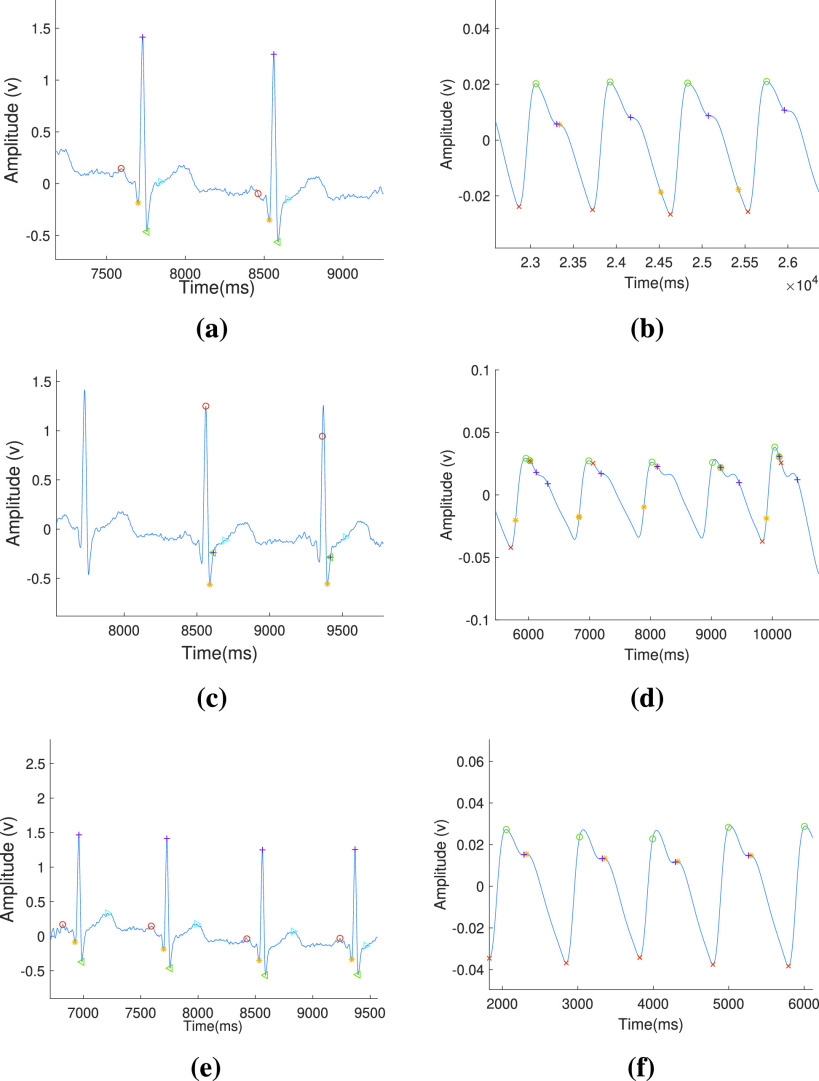
Comparison of wave point detection in Daubechies (a) ECG (b) PPG, Symlets (c) ECG (d) PPG, and Haar (e) ECG (f) PPG.

DWT is performed using Daubechies (Db), Symlets (sym), and Haar wavelets. Daubechies and Symlets are continuous wavelets and have a morphology similar to the ECG signal. Haar wavelet is the simplest function which involves a transition from ‘+1’ to ‘−1’ at 50% of its duty cycle. The comparison of the performance while wave point detection is shown in Fig. 1. It can be observed that the detection of some PPG wave point is performed more accurately with the help of the Db wavelet, whereas Haar performs more accurately in the case of ECG. The reason might be the constraints imposed within the algorithm for ECG wave point detection. Sym, on the other hand, is not performing as well as Db or Haar. The accuracy of detection using each wavelet has been compared in section III-B.

Therefore, due to low computational complexity and its ability to detect the sudden change within the signal, the proposed algorithm uses the Haar function for wavelet transformation. Despite having some drawbacks such as discontinuity and being non-differentiable, it is good enough to solve the purpose here. Along with DWT, Modulus Maxima Analysis (MMA) is performed, which helps in locating singularities within the signal. Feature extraction has been performed on a pulse of ECG signal by Mazomenos *et.al.* [25] using DWT and MMA. The process of feature detection and extraction is discussed further.

### C. Wave Point Detection on ECG Waveform

ECG signal is a physiological signal denoting electrical activities of the heart. Conventional ECG signals are obtained using 10 electrodes and 12 leads. These electrodes are placed at different points to obtain the waveforms describing the functioning of various parts of the heart. The 12 lead waveforms obtained are I, II, III, AVR, AVL, AVF, V1, V2, V3, V4, V5, and V6. Out of these, lead II waveform, also known as sinus rhythm is used here for this analysis. It is because of the proximity of the signal vector to that of the heart vector. Every cardiac cycle contains P, Q, R, S, T, and U waves. These waves symbolize various activities of the heart during depolarization and repolarization. The wavelet transform is used to distinguish various frequency components within a cycle and detect these waves, which here are called wave points.

**FIGURE 2.**
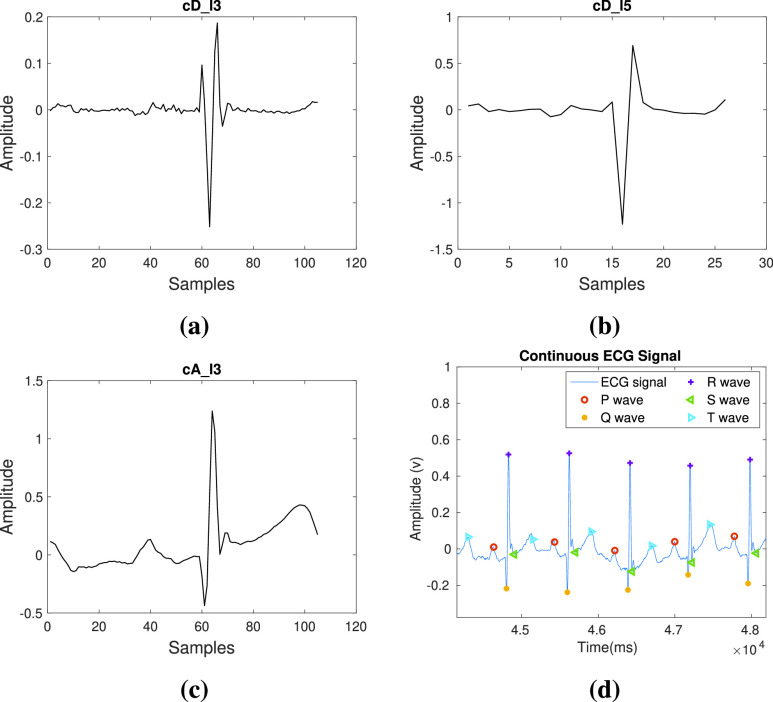
The plot of coefficients of wavelet analysis of single ECG cycle: (a) third level deterministic coefficients, (b) fifth level deterministic coefficients, (c) third level approximated coefficients and (d) detection of features in continuous ECG signal.

Pseudocode for ECG wave point detection is presented in Algo. 1, where Q, R, and S waves are detected using third-level wavelet transformation. One ECG pulse is considered in a window for analysis. Fig. 2a and 2c shows third level deterministic coefficients $cD\_{}l3$ and third-level approximated coefficients $cA\_{}l3$ of a single ECG pulse respectively, which are used for estimation of QRS complex. Among the sample points of $cD\_{}l3$, the minimum and maximum amplitude points are detected and termed as $t_{1}$ and $t_{2}$. Deterministic coefficients represent the derivative i.e. slope of the signal. Therefore, the R peak is detected as the maximum point in the ECG window between $t_{1}$ and $t_{2}$. MMA is performed on the ECG window and $t_{3}$ and $t_{4}$ are calculated as the modulus-maxima before $t_{1}$ and after $t_{2}$ respectively. Q peak is detected as a minimum amplitude point between $t_{3}$ and R peak. Similarly, the S peak is detected as a minimum amplitude point between R peak and $t_{4}$. Threshold values are set experimentally to detect the QRS complex. While moving from R peak towards the beginning, the first point where the differentiated value exceeds the first threshold is detected as QRS beginning and the last point from R peak towards the end of the differentiated signal that exceeds the second threshold is detected as QRS complex end.

**Algorithm 1:**
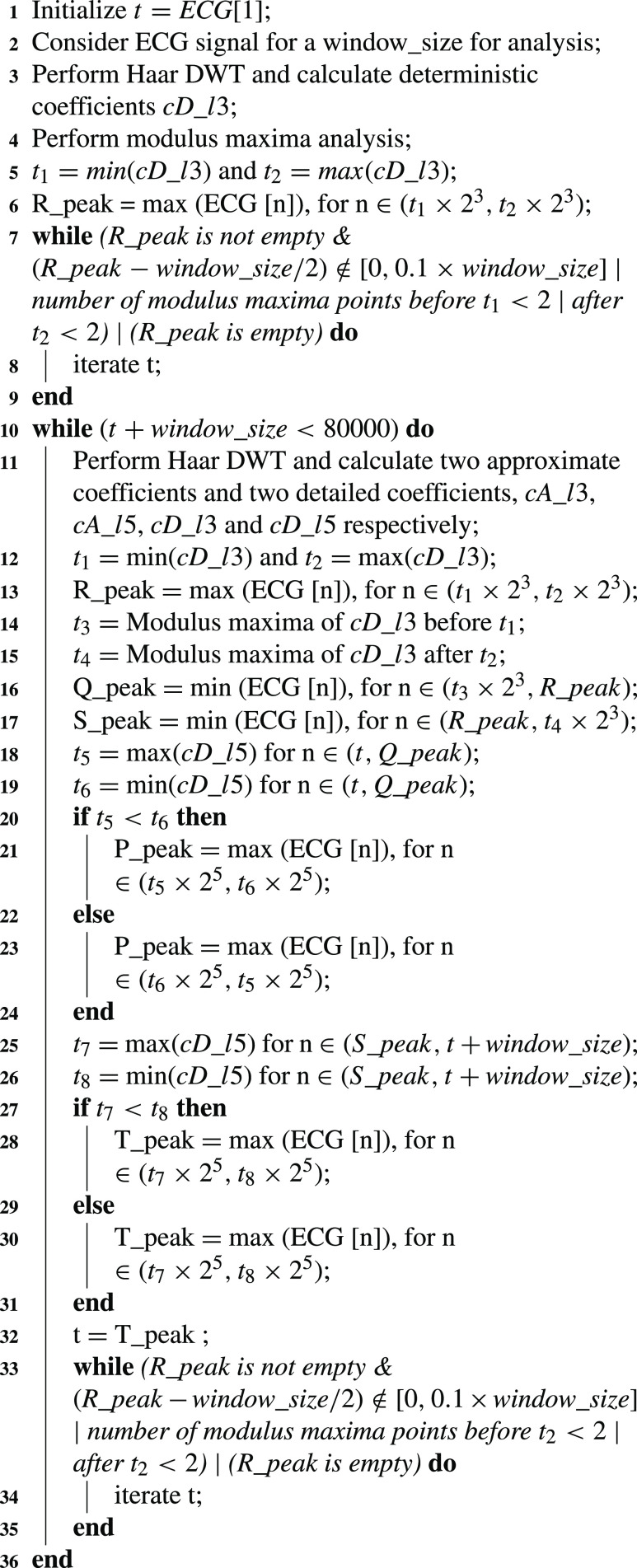
Pseudocode for ECGWave Point Detection

However, for detection of P peak and T peak, fifth level wavelet transformation is required. This is because, P and T waves are lower frequency waves in comparison to the QRS complex, and thus, their estimation is better at this level. The fifth level deterministic coefficients as shown in Fig.2b are used for the detection of the P wave and T wave. The fifth level waveform from the beginning of the window to the beginning point of the QRS complex is considered for the detection of the P wave. Similarly, T wave is detected as the point of maxima between the maximum and minimum points among the fifth level deterministic coefficients while considering points from QRS complex ending to the end of the window.

In this way, all the above stated fiducial wave points are detected out of a single ECG pulse. Now, the challenge is to detect these points within a continuous signal. For this purpose, the detection of the beginning of each ECG pulse is necessary to have a complete cycle within the window every time. The window is defined as the set of sample points in consideration for wave point detection, and its size is termed as window_size.

To have a complete pulse within a window in the required pattern, the below-listed conditions must be satisfied.

• R peak must be detected
• R peak should be in the vicinity of 10% of the window size about the center of the window
• Number of modulus maxima points (MMT) before and after R peak should be greater than 2

The presence of two MMT points before and after R peak ensures the presence of P wave and T wave inside the window respectively. If the above conditions are not satisfied, then the window is slid by 10 sample points to check for the presence of a complete pulse inside the window. Thus after every iteration of wave point detection, the beginning of another pulse is found by starting from the ending of the T wave inside the current window. In this way, necessary points are detected in continuity as shown in Fig. 2d.

### D. Wave Point Detection on PPG Waveform

PPG uses light-based technology to detect the changes in the blood volume. This is achieved using two techniques: reflection-based and transmission-based techniques [38]. In a single PPG cycle, we observe a peak and a notch. In some PPG devices notch is observed before the peak. Here the analysis is done on the data collected from the transmission based PPG device, where peak represents diastole. The detected light intensity is lesser in case of systole i.e. more absorption (due to the presence of high blood volume) and more in the case of diastole. Thus, the variation in the blood volume is conveyed by the PPG signal.

**FIGURE 3.**
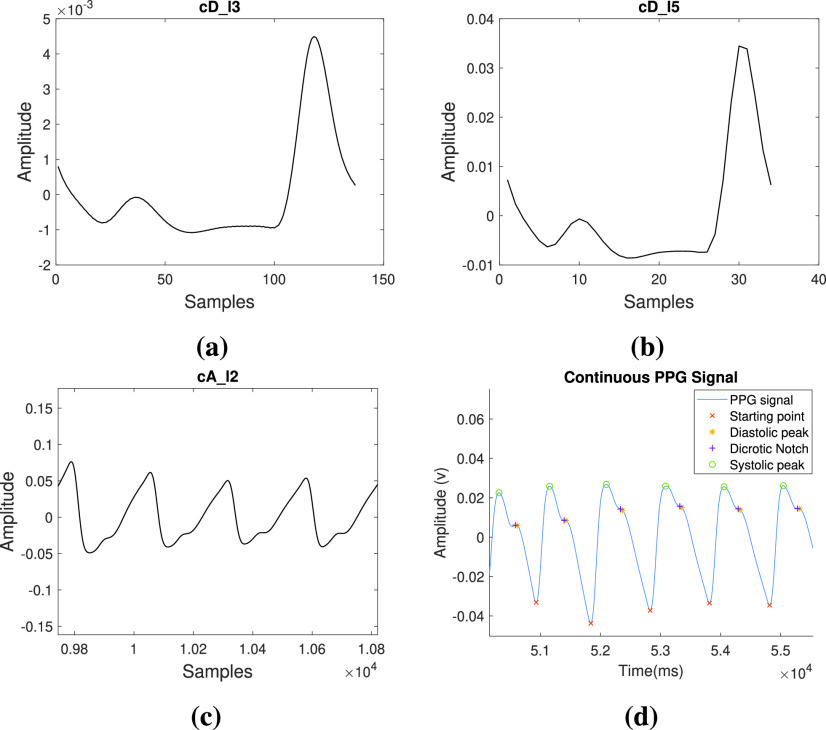
The plot of coefficients of wavelet analysis of single PPG cycle: (a) third level deterministic coefficients, (b) fifth level deterministic coefficients, (c) the second level approximated coefficients for continuous PPG signal and (d) detection of features in continuous PPG signal.

In each pulse of PPG waveform, Diastolic Peak (DP) and End of the Beat (EB), Systolic Peak (SP), and Dicrotic Notch (DN) have been detected and these points are termed as wave points in this case. It is accomplished using WT and Modulus Maximum techniques (MMT). To detect the peak as discussed in pseudocode in Algo. 2, third level wavelet transform is performed on the raw single PPG pulse as shown in Fig. 3. Raw here refers to pre-processed PPG waveform in which the notch appears before the peak. $t_{1}$ and $t_{2}$ are detected in the same manner as in case of ECG feature extraction discussed above. EB is detected between $t_{1}$ and $t_{2}$ as the maximum amplitude point. For the detection of the DP, high-level artifacts are removed to avoid the wrong detection. To achieve this, the fifth level coefficients (Fig. 3b) of the PPG waveform are obtained and differentiated. The first zero-crossings observed in the differentiated signal while moving towards left from the maximum point of the differentiated wave is detected as the DP. The beginning of the next pulse is detected using the EB of the current pulse.

**Algorithm 2:**
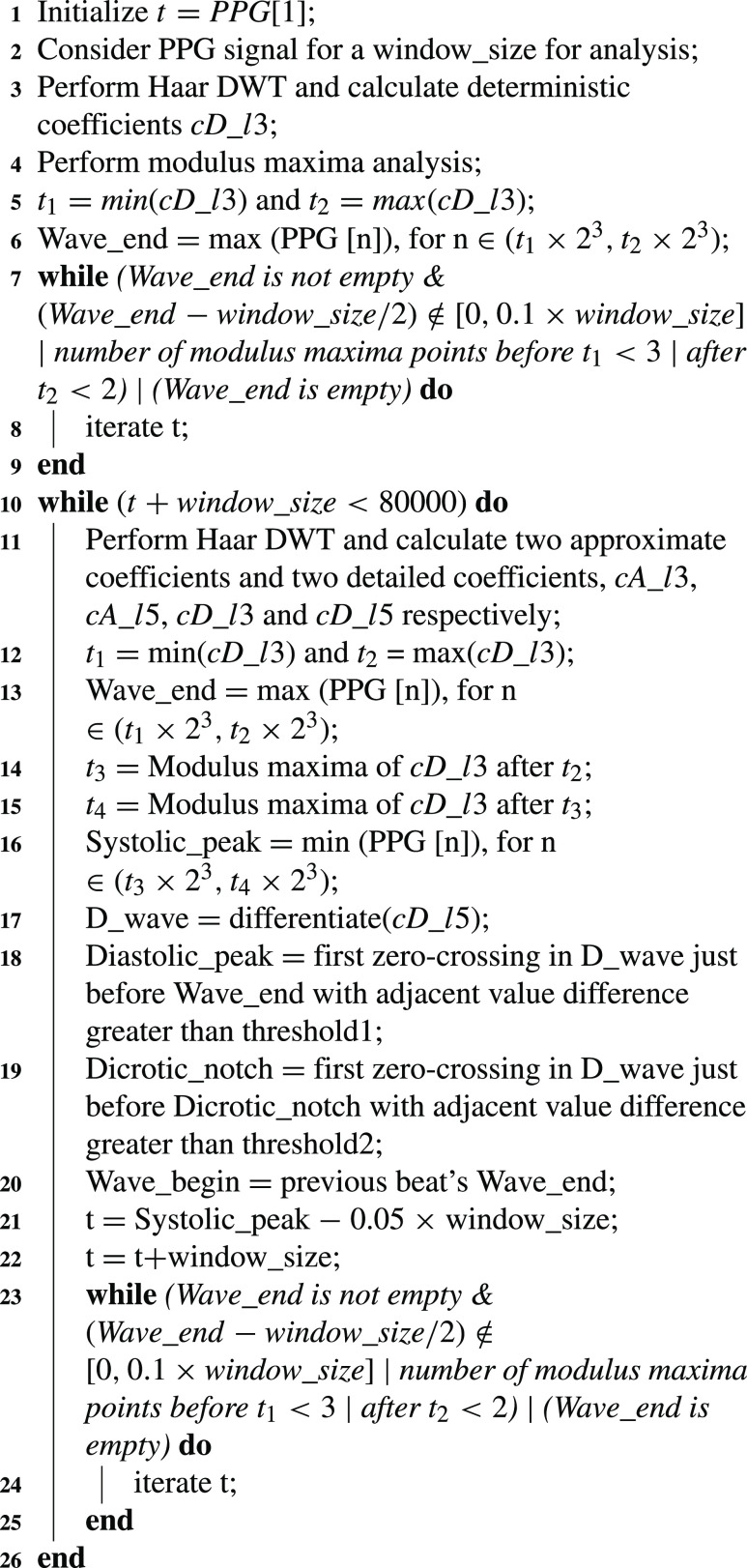
Pseudocode for PPG Wave Point Detection

For the detection of these points in a continuous PPG signal, we will always begin the window from the sample point one-tenth the size of the window before the detected wave beginning. This is done to ensure the presence of a complete pulse inside the window. Further to detect the beginning of a pulse and to check for the presence of the above-mentioned PPG wave points, samples are iterated unless the following conditions are satisfied.

• EB must be detected
• EB peak should be in the vicinity of 10% of the window size about the center of the window
• Number of modulus maxima points (MMT) before EB should be greater than 3 and after EB peak should be greater than 2

The presence of 3 MMT points before EB ensures the presence of DN and SP, and the presence of 2 MMT points after EB ensures the presence of SP inside the window. If the window does not satisfy the above conditions, it is shifted and this process is repeated until the first cycle having wave points in the required order is detected. In this way, wave points are detected on the continuous processed PPG signal as shown in Fig. 3d.

### E. Feature Detection

Techniques used for wave point detection are Fixed Window Wavelet Transform (FWWT), Adaptive Window Derivative, and Thresholding technique (AWDT) and finally, Adaptive Window Wavelet Transform (AWWT). FWWT uses a fixed window size of 1100 samples i.e. had an HR limitation of 52 to 98 beats per minute. The next window is obtained by considering the next 1100 samples. Hence, it is unable to check the availability of complete beat within the window i.e. for lower HR, there may not a complete beat and for higher HR, there may be more than one beat within the window. On the other hand, AWDT involves adaptive window size calculation, but, to reduce the time consumed for wavelet transformation, derivatives are calculated for the waveform in the window, and wave points are detected by comparing to the thresholds, calculated empirically. This technique helped in faster and adaptive detection, but the accuracy is compromised. Hence, the proposed algorithm performs wave point detection upon the continuous signals using AWWT. The comparison is made between these techniques, which are discussed in section III-A. The proposed technique of AWWT uses adaptiveness while calculating window_size, which gives an edge to this research. It enables precise detection of wave points and accurate extraction of the features. The window_size of the ECG signal is heart-rate dependent and that of PPG signal is pulse-rate dependent. Heart-rate is defined as the number of beats per minute, whereas, pulse-rate is the number of extensions in an elastic artery, that is, it is related to the hemodynamics of the body. Typically, heart-rate is equal to the pulse-rate under normal conditions, but for patients with cardiac problems, it may be different due to weaker heartbeat or inability to feel the pulsation. To estimate the window_size for ECG wave point detection, we first detect the R peaks using threshold technique applied on 2400 samples of the ECG signal, which is double the size of the sampling rate. These samples can accommodate only 2–3 R peaks. The distance between two adjacent R peaks is calculated and is considered as the size of the adaptive window. Similarly, the distance between two consecutive diastolic peaks is calculated for the size of the adaptive window to detect PPG wave points. As this size keeps on varying, it is calculated after each iteration. Also, some buffer is added to the calculated window size to ensure the presence of a complete pulse within a window.

### F. Feature Extraction and Error Handling

Features are extracted from the matrices formed during wave point detection. For statistical analysis, features are extracted from 80000 sample points, which is approximately sample points collected for a minute. A normal resting heart rate ranges from 60 to 100 beats per minute i.e. 1 to 1.6 beats per second. Therefore, around 60 to 100 beats have been utilized (based on heart rate).

31 features in addition to PAT are calculated from the detected ECG and PPG wave points. It can be concluded based on the references [6], [10], [12]–[14], [16], [20], [23], [24], that use of extra features along with PAT, results in an improvement in accuracy of BP estimation. Hence, in this work, we included the features suggested in the prior works, along with some of the addition similar features based on the fact that PATs represent the duration between various activities of the heart and the instants their effects are observed in the periphery. These features have been listed below. The attempt is to extract all the possible features out of ECG and PPG signal, to check their variance and correlation with BP.

The list of the 32 features is as follows:

• Tqrs (QRS Complex length)
• Tpr (PR interval)
• Tst (ST interval)
• Trr (RR interval)
• Tpt (PT interval)
• Tab (EB-DP interval)
• Tbb (EB-EB interval)
• Taa (DP-DP interval)
• Tn1b (DN-EB interval)
• Tn2b (SP-EB interval)
• Tn2n1 (SP-DN interval)
• Tn2a (SP-DP interval)
• Tia (duration between i peak and DP)
• Tib (duration between i peak and EB)
• Tpn (duration between i peak and DN)
• Tin2 (duration between i peak and SP)
  ’i’ here stands for P, Q, R, S and T peaks of ECG cycle

**FIGURE 4.**
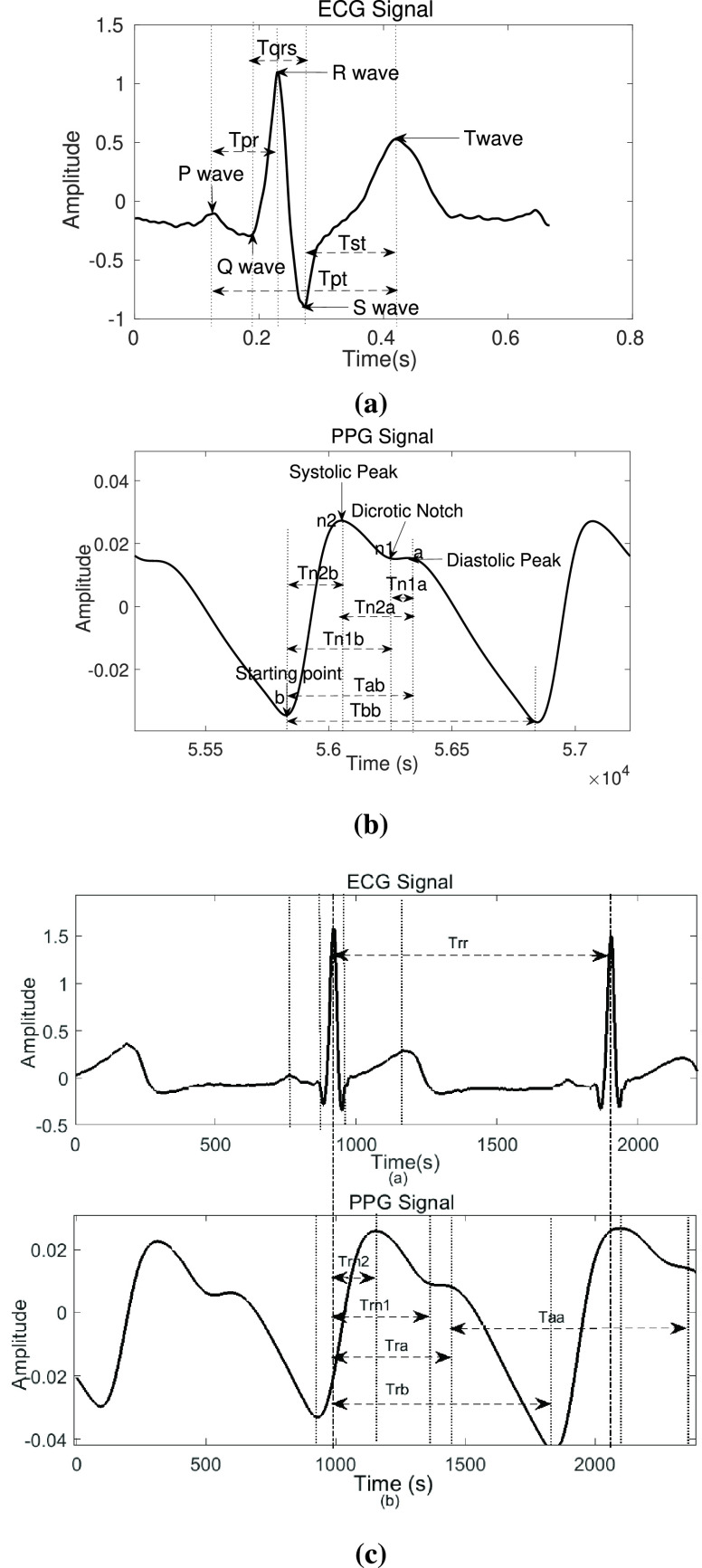
Extraction of features from (a) ECG signal, (b) PPG Signal and (c) ECG signal and PPG Signal simultaneously.

Among the 32 features, 20 features are obtained from a comparative analysis of the ECG and PPG wave points as shown in Fig. 4c. These features involve the difference between each of the 5 wave points of the ECG signal and each of the 4 wave points of the PPG signal. These are the transit times calculated similarly to PAT. 5 features are calculated solely from the ECG wave points. These features i.e. P-R, QRS, S-T, P-T, and R-R interval as shown in Fig. 4a, are the lengths of ECG beat segments that inform about repolarization and depolarization of the heart during systole and diastole. The rest of the 7 features are calculated from the PPG wave points exclusively. These involve EB-DP, EB-EB, DP-DP, EB-DN, EB-SP, DN-DP and SP-DP interval as shown in Fig. 4b. They represent the blood volumetric variations in the periphery during systole and diastole.

**FIGURE 5.**
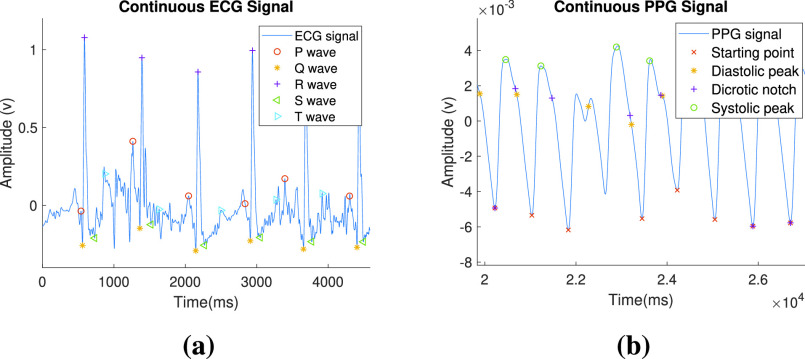
Detection in a bad beat for (a) ECG, and (b) PPG waveform.

There are several other measures taken to handle the bad beats apart from the conditions discussed in Algo. 1 and Algo. 2 for ensuring a complete beat within the window. The wave point detection for a bad beat in the case of ECG and PPG waveform is shown in Fig. 5a and 5b respectively. To ensure that features are calculated only among the detected points of the simultaneous pulses of ECG and PPG signal, R peak is back-traced from the time instant at which DP is detected for a particular pulse. The index of that DP is used for all the matrices in the PPG signal and the index of that R peak is used for all the matrices of ECG signal. Mean and SD are calculated for each feature’s matrix and to account for errors during detection, all the values lying out of the range of [mean − SD, mean + SD] are removed. An average is calculated for the remaining values, which is considered as the feature value in the feature vector. Regression analysis is performed on the created feature vector to train a model that can be used to estimate the SBP and DBP. Regression models have been discussed ahead in the next subsection.

### G. Regression Model

In the prior research works, several machine learning techniques have been used for BP modeling. Some among those are linear regression [23], [24], [26], [39], non-linear regression [27], neural networks [11], [21], etc. The proposed algorithm uses the log-linear model for training. Log-linear is a semi-log model in which the log of the dependent variable is equal to the linear combination of the weighted independent variables and a constant. The intuition behind using this model is the inverse-exponential relation of PTT with BP. Also, the independent features, that is, the values of input features are of the order of 10^−3^, whereas the dependent variables are of higher order comparatively. The effectiveness of log-linear approach modeling is proven empirically by observing the residuals i.e. (expected-observed). The residuals obtained in this case were lower than all the other techniques as discussed in the further section.

## III. Results and Validation

The accuracy of the estimation of BP using various techniques is measured and compared in terms of Mean Absolute Error (MAE), Mean Error (ME) and Standard Deviation (SD), defined by [31] as equations (3), (4) and (5), where $p_{i}$ is the estimated value, $y_{i}$ is the reference value and $n$ is the data size. The error calculated by the comparison of estimated BP to the reference BP is used for statistical analysis performed using designated functions in MATLAB.

\begin{align*} ME=&\left({\sum _{i=1}^{n}p_{i} - y_{i}}\right)/n, \tag{3}\\ MAE=&\left({\sum _{i=1}^{n}|p_{i} - y_{i}|}\right)/n, \tag{4}\\ SD=&\sqrt {\left({\sum _{i=1}^{n }((p_{i} - y_{i})-ME)^{2}}\right)/n-1} \tag{5}\end{align*}

### A. Statistical Analysis

Acquisition of synchronized ECG and PPG data is performed for all subjects in a sitting position. The variation observed in terms of mean and SD within acquired reference SBP values is 128.45 ± 15.34 mmHg, whereas, in the case of DBP, it is 77.73 ± 9.16 mmHg. The ECG and PPG data used in the algorithm have a common sampling rate of 1200 samples/sec. Sample points after 10000 points are considered, as the PPG signal takes some time to settle. Thereafter, extracted features are averaged over a minute to evaluate intermittent BP for performing statistical analysis.

**FIGURE 6.**
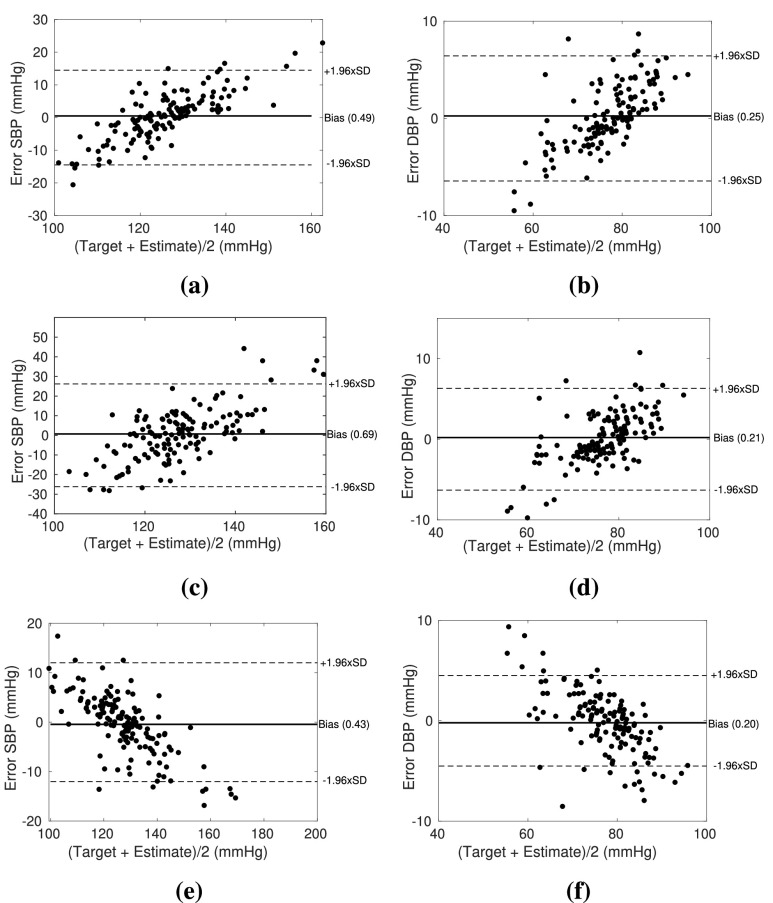
The Bland-Altman plot for (a) FWWT SBP (b) FWWT DBP (c) AWDT SBP (d) AWDT DBP (e) AWWT SBP (f) AWWT DBP estimation.

The errors obtained using FWWT are 0.49 ± 6.39 mmHg and 0.25 ± 3.28 mmHg for SBP and DBP respectively. This can be visualized in Bland-Altman plots in Fig. 6a and Fig. 6b. The mean absolute error is 5.62 and 2.56 mmHg respectively for SBP and DBP. This method worked well with the SBP values lying in the range of around 121 to 140 mmHg but the error observed was high in the case of SBP out of this range, especially, higher than 140 mmHg i.e. hypertensive range. This also supports the correlation established between BP and HR by Matsumura *et.al.* [40]. Thus, to overcome this limitation of HR, the concept of an adaptive window is used. To reduce the time consumed by wavelet transformation, derivative and thresholding technique termed as AWDT was also used for detection. The errors as shown using the Bland-Altman plot in Fig. 6c and Fig. 6d for this technique are 0.69 ± 13.36 mmHg and 0.214 ± 3.21 mmHg for SBP and DBP respectively. Comparatively, the best results are obtained for AWWT. MAE of 4.6 and 2.3 mmHg and ME ± SD of 0.43 ± 6.13 and 0.20 ± 3.06 mmHg is obtained for SBP and DBP respectively. AWWT circumvents the shortcomings of both the two prior techniques i.e. FWWT and AWDT.

ANOVA (Analysis of variation) is performed to statistically evaluate the three techniques. It is performed to evaluate, how significant is the difference between these techniques. To do so, MAE calculated for SBP and DBP of all the 171 patients for each of these techniques is considered. Table 2 and Table 3 shows the Analysis. We can observe that F (ratio of the mean squares) and P-value (the probability that the F-statistic can take a value larger than the computed test-statistic value) obtained in SBP analysis is as per the expectation. Hence, for SBP, we can conclude that there is a significant difference between the techniques. On the other hand, for DBP, we can observe that the difference is not that significant.

**TABLE 2:** ANOVA for MAE Obtained While SBP Estimation

Source of Variation	SS	df	MS	F	P-value	F crit
Between	2502.542	2	1251.271	32.785	0.7 * 10^−13^	3.019
Groups Within	14846.41	389	38.16558			
Groups Total	17348.95	391

**TABLE 3:** ANOVA for MAE Obtained While DBP Estimation

Source of Variation	SS	df	MS	F	P-value	F crit
Between	8.183	2	4.0915	1.157	0.315	3.018
Groups Within	1387.3	392	3.539			
Groups Total	1395.4	394				

### B. Accuracy of Feature Detection

To calculate the accuracy of feature detection, ECG and PPG waveforms from 137 subjects have been taken into consideration. Reference wave points are marked on both the waveforms [28]. This is done for 12000 sample points i.e. 10 seconds of data, which is 10 to 16 beats. Wave points are detected using the algorithms discussed above (Algo. 1 and Algo. 2). Accuracy is henceforth calculated as the mean of the comparison of the obtained wave points with the reference ones. The achieved accuracy for wave point detection using wavelets, viz., Db, Sym, and Haar, is compared using Table 4. Based on results, it can be inferred that wave points are detected using Haar wavelet with sufficient accuracy to proceed forward with feature extraction.

**TABLE 4:** Quantitative Comparison of Wave Point Detection Using Different Wavelets

Wavelet	ECG					PPG			
	P	Q	R	S	T	EB	SP	DN	DP
Db	0.6	0.78	0.89	0.76	0.54	0.82	0.8	0.96	0.67
Sym	0.48	0.52	0.7	0.68	0.32	0.63	0.57	0.61	0.49
Haar	0.82	0.83	0.98	0.78	0.8	0.8	0.75	0.965	0.96

### C. Comparison of Features

The extracted features are evaluated using feature reduction and feature selection techniques. Principle Component Analysis (PCA) is used for feature reduction. $R^{2}$ values are calculated. Higher the ‘$R^{2}$’ value, more will the variance in the feature. Around 15 features are found with values higher than 0.3, which are considered further for estimation. Thereafter, for feature selection, the wrapper technique is used. In this technique, features were selected randomly and the model was trained recursively. Based on the performance of the trained models, 9 and 14 features are selected for the estimation of SBP and DBP respectively. Hence, the number of features to be used is drawn experimentally based on both, the results obtained from PCA as well as the accuracy of the trained models.

The 9 features used for SBP estimation include P-DN, P-EB, R-DN, R-EB, P-R, Q-R-S, S-T, P-T and R-R interval. The 14 feature used for DBP estimation includes DN-EB, SP-DN, SP-DP, SP-EB, and EB-EB interval, in addition to the features used for SBP estimation.The contribution of each of these features to estimate SBP and DBP are discussed in Table 5 respectively.

**TABLE 5:** Features for SBP and DBP Estimation (X Stands for Each Feature and Y Stands for Dependent Variable)

S.No	Feature	SBP		DBP	
		% var in x	%var in y	% var in x	% var in y
1	P-DN	0.810	0.051	0.708	0.044
2	P-EB	0.038	0.107	0.083	0.047
3	R-DN	0.046	0.046	0.057	0.025
4	R-EB	0.037	0.054	0.036	0.038
5	P-R	0.052	0.010	0.059	0.014
6	Q-R-S	0.006	0.007	0.029	0.013
7	S-T	0.005	0.001	0.008	0.006
8	P-T	0.005	0.005	0.007	0.003
9	R-R	0.001	0.001	0.004	0.002
10	DN-EB			0.002	0.002
11	SP-DN			0.003	0.003
12	SP-DP			0.002	0.002
13	SP-EB			0.001	0.001
14	EB-EB			0.003	0.003

### D. Comparison of Regression Techniques

The various regression analysis has been performed on the features calculated using AWWT, to obtain a model for BP estimation. The dimension of the feature vector is high, therefore, there was a requirement of a large amount of data for the training purpose. The results for SBP and DBP models are summarized in Table 6.

**TABLE 6:** Performance Analysis of Estimation of SBP and DBP Using Various Regression Techniques

Statistical Parameters		Multiple Linear	Rational Quadratic	Exponential	Square-exponential
SBP	RMSE	0.06	0.078	0.10	0.044
	MAE	0.041	0.060	0.051	0.054
	R-Squared	0.48	0.46	0.39	0.21
DBP	RMSE	0.061	0.089	0.68	0.078
	MAE	0.056	0.051	0.045	0.050
	R-Squared	0.49	0.33	0.61	0.29

### E. Validation Using Phase II Data

Data for 28 subjects was collected using the same procedure as discussed in section II-A. This was done to validate the regression models obtained for SBP and DBP. These subjects belong to the age-group of 36 ± 10 years. The variation in SBP and DBP is 127 ± 19 and 76 ± 15 mmHg respectively. The estimation accuracy obtained is 0.3 ± 3.38 mmHg and 0.57 ± 1.9 mmHg for SBP and DBP respectively. MAE obtained is 2.76 and 1.98 mmHg respectively. Hence, the regression model for BP estimation is validated as discussed in the subsection below.

## IV. Discussion

In this study data has been collected as per IEEE Standard 1708 [31]. The minimum need of subjects within various data ranges have been met. [31] is compliant to British Hypertension Society (BHS) [41], as well as AAMI standards [42]. BHS standards are based on cumulative error percentages, whereas AAMI standards are based on ME and SD values. The comparison can be seen in Table 7 and 8 respectively.

**TABLE 7:** Comparison With BHS Standards Based on Cumulative Error Percentage

		≤ 5 mmHg	≤ 10 mmHg	≤ 15 mmHg
This work	SBP	63.7	87.9	98.6
	DBP	87.9	100	100
BHS [41]	grade A	60%	85%	95%
	grade B	50%	75%	90%
	grade C	40%	65%	85%

**TABLE 8:** Comparison With AAMI Standards Based on Statistical Parameters

		ME (mmHg)	SD (mmHg)	Subjects
This work	SBP	0.43	6.13	171
	DBP	0.20	3.06	171
AAMI [31]	SBP and DBP	≤ 5	≤ 8	≥ 85

Based on this comparison, the following can be inferred about our results:

• grade A as per BHS standards
• qualify AAMI standards

Hence, as per IEEE Std 1708 [31] for cuffless blood pressure measuring device, which also considers BHS and AAMI standards, the recommended grade is ‘A’

## V. Conclusion

An adaptive algorithm (AWWT) has been introduced in this paper for continuous and real-time estimation of SBP and DBP using multiple features extracted from concurrent ECG and PPG signals using Haar transformation. It attenuates the noise adaptively. It not only ensures precise detection of the wave points but also adds robustness towards heart-rate and pulse-rate variability. In the future, these features can also be used in the detection of tachycardia, bradycardia, arrhythmia, etc. For dimension reduction, PCA was used and consequently, the features for SBP and DBP are selected based on the obtained accuracy while training the model. The model is trained by using log-linear regression analysis on an extensive feature vector formed by performing wave point detection within the ECG and PPG data collected as per guidelines [31]. As a resultant, we observe the training accuracy of 0.43 ± 6.13 and 0.20 ± 3.06 mmHg and MAE of 4.6 and 2.3 mmHg for the estimation of SBP and DBP respectively. The model was also tested on the un-trained data to ensure the reliability of the model. The results obtained are as per the AAMI and BHS standards [41], [42]. Henceforth, this algorithm can be used for continuous BP measurement in the next generation portable integrated system of ECG and PPG measurement.
